# Personalized Contrast Agent Volumes in Abdominal CT: Bridging Theory with Practice

**DOI:** 10.5334/jbsr.3906

**Published:** 2025-06-09

**Authors:** Walter Coudyzer, Lesley Cockmartin, Bram Miseur, Tim Busselot, Didier Bielen, Dirk Vanbeckevoort, Raymond Oyen, Hilde Bosmans

**Affiliations:** 1 Department of Radiology, University Hospitals KU Leuven, Herestraat 49, 3000 Leuven, Belgium

**Keywords:** abdominal CT examinations, patient tailored contrast injections, iodinated contrast agents, contrast agent scan delays, contrast volume calculation, HU target values

## Abstract

*Objectives:* to determine (1) a target Hounsfield unit (HU) for portal venous phase iodinated contrast media enhanced abdominal CT scans, (2) an equation for a personalized total contrast agent volume, and (3) the best/most appropriate time between injection and plateau/peak in HU enhancement.

*Material and methods:* from an original dataset of 5,000 cases, a weight representative subset of 370 cases was sampled for detailed HU measurements. An additional 90 cases were used for visual grading to define the minimal HU required for diagnostic quality, which led to the proposed target HU. This study uses the fact that in a first approach, the injected contrast agent volume and HU correlate linearly. Based on the injected contrast agent volumes and HU measurements in the patient scans, it was then calculated which (ideal) volume would have reached the target value. The ideal volumes and patient data (weight, height, heart rate, age, and gender) were correlated by means of a regression analysis, to determine a new patient-specific contrast volume calculation equation. The best scan delay time was derived from the start of the injection to the HU enhancement plateau/peak evaluated from manually triggered venous phase scans.

*Results:* The target HU value was 125. This can be achieved with a personalized contrast agent volume (ml), equal to − 108.5 + ∗ *weight(kg)* + 0.40 ∗ *heart rate(bpm)* + 0.61 ∗ *height(cm)*. The time delay between injection and HU plateau/peak was found to be, on average, 102 s.

*Conclusion:* this study proposes a comprehensive protocol for contrast enhanced venous phase scans, including a target HU, a personalized contrast volume, and a scan delay.

## Introduction

A large number of abdominal CT scans are performed with intravenous iodinated contrast media to assess perfusion in vessels, parenchymal structures, and lesions. The portal venous phase is the most frequently utilized, differing from arterial phase scans as it visualizes tissue perfusion several seconds after the contrast bolus arrival in the arteries [1]. Unlike arterial scans, the tracking of contrast arrival in the parenchyma is not yet standard in current CT technology [2–4]. Instead, portal venous imaging typically employs fixed scan initiation times post‑injection [5]. The constant trigger delay times are historical. We are not aware of a verification of these times for the newer generation of (faster) CT scanners. Conversely, historical contrast agent volumes have been addressed with more individualized methods, such as those based on patient weight, lean body weight (LBW) or body surface area (BSA). A few publications have shown more stable contrast enhancement, expressed via Hounsfield unit (HU) values in different organs [6–16]. These personalized approaches offer a way to standardize contrast enhancement across patients, paving the way for a more reliable diagnosis. However, the current literature lacks a unified framework or evidence‑backed guidelines for achieving optimal enhancement. Many equations for tailored injection schemes, including those used in our hospital, are pragmatic but not rigorously validated.

The innovative aspect of our study lies in its comprehensive, data‑driven approach to optimizing contrast agent administration. By leveraging retrospective data, we aim to address the limitations of existing practices. Diagnostic image quality with minimized risks of adverse effects is the target [17, 18]. Our emphasis on achieving uniform tissue enhancement across patients should help ensure that variations in HU reflect pathological conditions rather than patient size or suboptimal imaging protocols.

In the present study, we aim for a comprehensive protocol with the following items:

1. *A target HU threshold*: Through visual grading of scans, we aim to identify an ideal HU value indicative of successful contrast enhancement. This target provides a measurable benchmark to standardize imaging quality.
2. *Personalized contrast agent volumes*: Using regression analysis, we will develop a formula to calculate patient‑specific contrast agent volumes necessary to achieve the defined HU threshold. This approach adapts contrast dosage to individual patient profiles, minimizing both under‑ and over‑enhancement.
3. *Optimized timing*: By manually initiating scans at the plateau or peak of HU enhancement post‑contrast agent injection, we will determine the optimal delay for initiating portal venous phase imaging.

The results of the present project will represent a transformative step towards precision imaging and evidence‑based injection protocols that may ultimately enhance both diagnostic accuracy and patient safety.

## Material and Methods

Data from 5,000 patients who underwent portal venous phase CT scans between 2015 and 2019 were analyzed from scans on four CT scanners (Siemens Somatom Force, Siemens Somatom Definition Flash, Philips Ingenuity, and Canon Aquilion One) using standardized protocols. Patient demographics (gender, age, weight, height) and heart rate were recorded, while contrast volumes were calculated using iCalc software (Medicor Int., Rotselaar, Belgium) based on BSA, heart rate and a fixed scaling parameter.

The iCalc formula is:


$$\begin{array}{rcl} Contrast\;volume\; & = & \;BSA\;\left(m^{2}\right)\;*\;C\\ & +\; & heart\;rate\;based\;volume\;corrections \end{array}$$ Eq. 1

with corrections as described in Table 1 and *C* = 45  ml/m² (based on Yanaga’s study [19]). In addition, calculated injected volumes were cut off to be never less than 40 ml and never exceed 120 ml.

**Table 1:** Adjustments to the injected volumes based on heart rate and iodine concentration.

HEART RATE (BPM)	INJECTED VOLUME CORRECTION (ML)
<55	− 10
56–65	+ 0
66–75	+ 10
76–90	+ 20
91–105	+ 25
>105	+ 30
Iodine concentration (mg I/ml)	
320	+ 8
350	+ 0
370	+ 0

The research comprised three experiments:

1. *Target HU values*
  The study identified target HU values for optimal venous enhancement using visual grading analysis (VGA) of 90 CT scans in part sampled from the 5,000 cases dataset and then further completed outlier cases in terms of HU, weight, and heart rate. Two radiologists rated the enhancement quality on a five‑point scale, focusing on hepatic veins, liver parenchyma, spleen, and kidneys. The number of cases was in part inspired by an early reference on reading studies for image quality scoring [20]. The target HU was set at the 25th percentile of the high‑quality cases (score ≥ 4) but then increased with a square root of 2[Bibr r2] or 1.4 times the standard deviation of the HU values present in the analysis of 370 cases (see further). With this target value and based on Chebyshev’s inequality, it is then ensured that at least 50% of the cases will have an HU higher than the 25th percentile. The target HU formula is:
$$\begin{array}{ccc} Target\;HU\; & = & \;25th\;percentile\;HU\;value\;of\\ & & cases\;with\;score\;4\;or\;higher\\ & & +\;1.4\;*\;standard\;deviation\;on\;the\;HU. \end{array}$$ Eq. 2
2. *Personalized contrast agent volumes*
  In a 370‑patient dataset, sampled from the 5,000 cases, ensuring an equal distribution of weights, the HU values were measured in seven organs (vena cava inferior, vena porta at the liver hilus, hepatic parenchyma segment 2, hepatic parenchyma segment 7, spleen parenchyma, left kidney cortex, and right kidney cortex), and the mean HU values of these organs were calculated. The study then examined the relationship between patient‑specific variables (weight, height, heart rate, age, gender) and contrast agent volumes, and calculated the ideal volumes that would have given the target HU value. A linear correlation between contrast agent volume and HU was assumed:
$$\begin{array}{ccc} ideal\;volume & = & original\;volume\\ & & +\;\frac{original\;volume}{{HU}_{measured}}\\ & & *({HU}_{target}-{HU}_{measured}) \end{array}$$ Eq. 3
  Regression analysis between the ideal volumes and several patient parameters yielded the new formula for personalized contrast volumes.
3. *Timing optimization for peak enhancement*
  A prospective study on 100 patients was performed to investigate the interval between contrast agent injection and peak tissue enhancement. *A posteriori*, it was shown that this number of cases led to a statistically valuable result. Using manual scan initiation, the researchers monitored HU levels at 2 s intervals in a liver region of interest (ROI) starting 30 s post‑injection. A second regression analysis was performed between the personalized intervals and the patient characteristics. The mean delay time between start of injection and peak or plateau enhancement was also calculated.

Prior to any regression analysis, HU measurements were corrected towards the HU value that would have been obtained if the scan had been performed with 120 kVp. In practice, if the scan was performed with 100 kVp, the HU values were reduced by 22%, while for 140 kVp scans, the predicted value for a 120 kVp scan would be 14% higher. These values followed from test object measurements at several kVp.

## Results

The main findings and insights are summarized in the following paragraphs.

**Figure 1:**
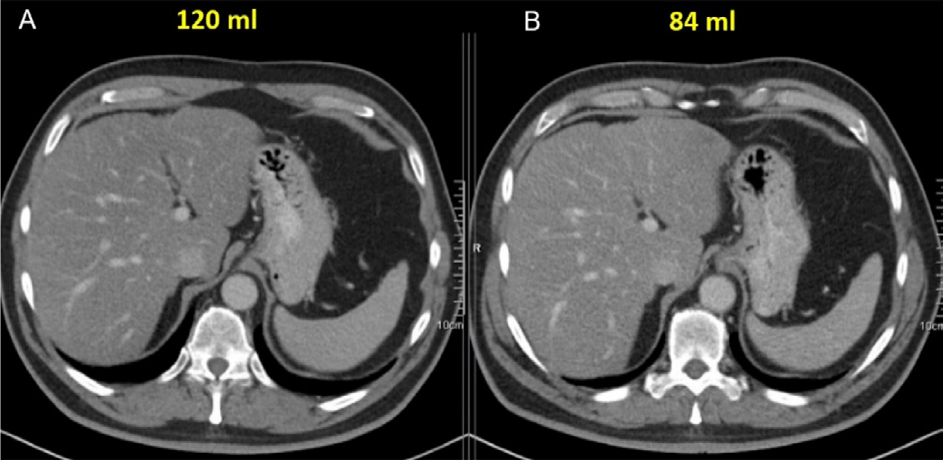
Portal venous phase contrast enhanced CT scans of the same patient. Oncology follow‑up patient. Scanned on two different scan dates (A & B) with an interval of six weeks. BMI = 31. **A**. Without contrast volume optimization (120 ml, VGA score 4). **B**. With contrast volume optimization (84 ml, VGA score 4).

Patient demographics and data:

The original dataset comprised 5,000 patients (2291 males, 2709 females). The men had received a mean contrast volume of 94 ± 17 ml, while the women received 79 ± 17 ml. The overall mean contrast volume was 86 ± 18 ml. Extra demographic data can be found in Table 2. Figure 1 illustrates two CT slices with and without contrast volume optimization and their corresponding VGA score.

**Table 2:** Descriptive statistics (mean, standard deviation (stdev), interquartile range (IQR)) of patient parameters for the total population of 5,000 patients, further subdivided for gender, as well as for the study dataset of 370 cases.

	TOTAL POPULATION		MEN		WOMEN		STUDY DATASET (*N* = 370)	
	MEAN ± STDEV	IQR	MEAN ± STDEV	IQR	MEAN ± STDEV	IQR	MEAN ± STDEV	IQR
Age (years)	63 ± 13	55–72	63 ± 14	56–73	62 ± 13	54–71	61 ± 14	53–71
Weight (kg)	71 ± 15	60–80	77 ± 14	68–85	65 ± 13	56–73	71 ± 15	61–80
Height (cm)	169 ± 9	162–175	175 ± 7	170–180	163 ± 7	158–168	170 ± 10	164–180
Heart rate (bpm)	76 ± 15	65–85	73 ± 15	63–82	78 ± 15	67–87	75 ± 14	64–84
BSA (m²)	1.7 ± 0.4	1.4–1.9	1.9 ± 0.4	1.6–2.1	1.5 ± 0.3	1.3–1.7	1.7 ± 0.4	1.5–2.0
BMI (kg/cm²)	25 ± 5	22–27	25 ± 4	22–27	25 ± 5	21–27	25 ± 6	21–27
Contrast volume (ml)	86 ± 18	73–99	94 ± 17	82–107	79 ± 17	67–90	86 ± 19	74–99
Iodine dose (g)	NA	NA	NA	NA	NA	NA	28.5 ± 7.5	24.3−34.0

### Target HU value

**Figure 2:**
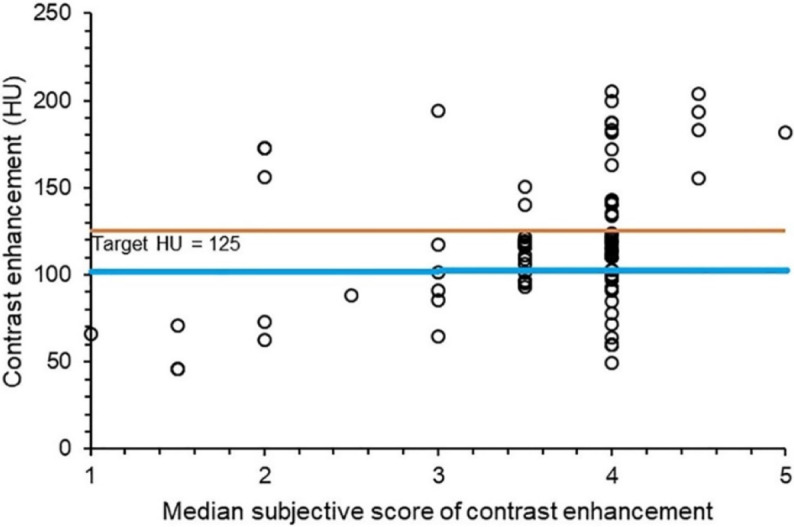
Objective HU measurements versus subjective ordinal performance score (median for three regions and two radiologists) for portal venous phase contrast enhanced CT abdomen scans. The blue line shows the 25th percentile that should be reached. The orange line shows the target of 125 HU that will ensure more than 90% of cases will achieve the 25th percentile.

VGA in a subset of 90 cases revealed acceptable contrast enhancement in 81% of scans, with a median VGA score of 4. The 25th percentile, increased with the square root of two times the standard deviation measured in the 370‑case dataset, was 125 (Figure 2).

Visual grading five‑point rating scale:

– 1 unacceptable: contrast enhancement insufficient to make a radiologic diagnosis
– 2 poor: contrast enhancement just about not acceptable to make a radiologic diagnosis
– 3 adequate: contrast enhancement just about acceptable to make a radiologic diagnosis
– 4 good: contrast enhancement sufficient to make a radiologic diagnosis
– 5 excellent: contrast enhancement sufficient to make a radiologic diagnosis

### Personalized contrast agent volumes

The subset of 370 patients, in which the HU values were measured, had a similar weight distribution as the larger cohort (Tables 2 and 3).

**Table 3:** Overview of scan parameters.

	SIEMENS FORCE	PHILIPS INGENUITY	SIEMENS FLASH	CANON AQUILION ONE
*n* patients	49	794	1284	2873
Ref. kV	120	120	120	120
Ref. mAs	170	150	170	180
Rot. Time (s)	0.5 s	0.5 s	0.5 s	0.35 s
Collimation	192 × 0.6 mm	64 × 0.6 mm	128 × 0.6 mm	40 × 0.5 mm
Pitch	0.6	1.49	0.6	0.5
Slice thickness	1 mm, 5 mm	1 mm, 5 mm	1 mm, 5 mm	1 mm, 5 mm
Slice increment	0.5 mm, 5 mm	0.5 mm, 5 mm	0.5 mm, 5 mm	0.5 mm, 5 mm
Kernel	abdomen/body	abdomen/body	abdomen/body	abdomen/body
Tube current modulation	CareDose 4D, CareKv	DoseRight, Z‑modulation, 3D‑modulation	CareDose 4D, CareKv	Aidr 3D
kV modulation*	100, 120	100,120	100, 120	100,120

*Calculated from a representative subsample (217 cases).

Regression analysis found significant relationships between the ideal contrast volumes and patient weight, height, and heart rate. Gender and age were not significant contributors in the regression analysis. Including then only the significant parameters (weight, height, and heart rate) in the next regression analysis resulted in the following formula to calculate the new (ideal) contrast volumes:


$$\begin{array}{ccc} New\;contrast\;volume & = & -108.5+0.97*weight(kg)\\ & & +\;0.40*heart\;rate(bpm)\\ & & +\;0.61*height(cm) \end{array}$$ Eq. 4

### Injection timing for plateau/peak enhancement

**Figure 3:**
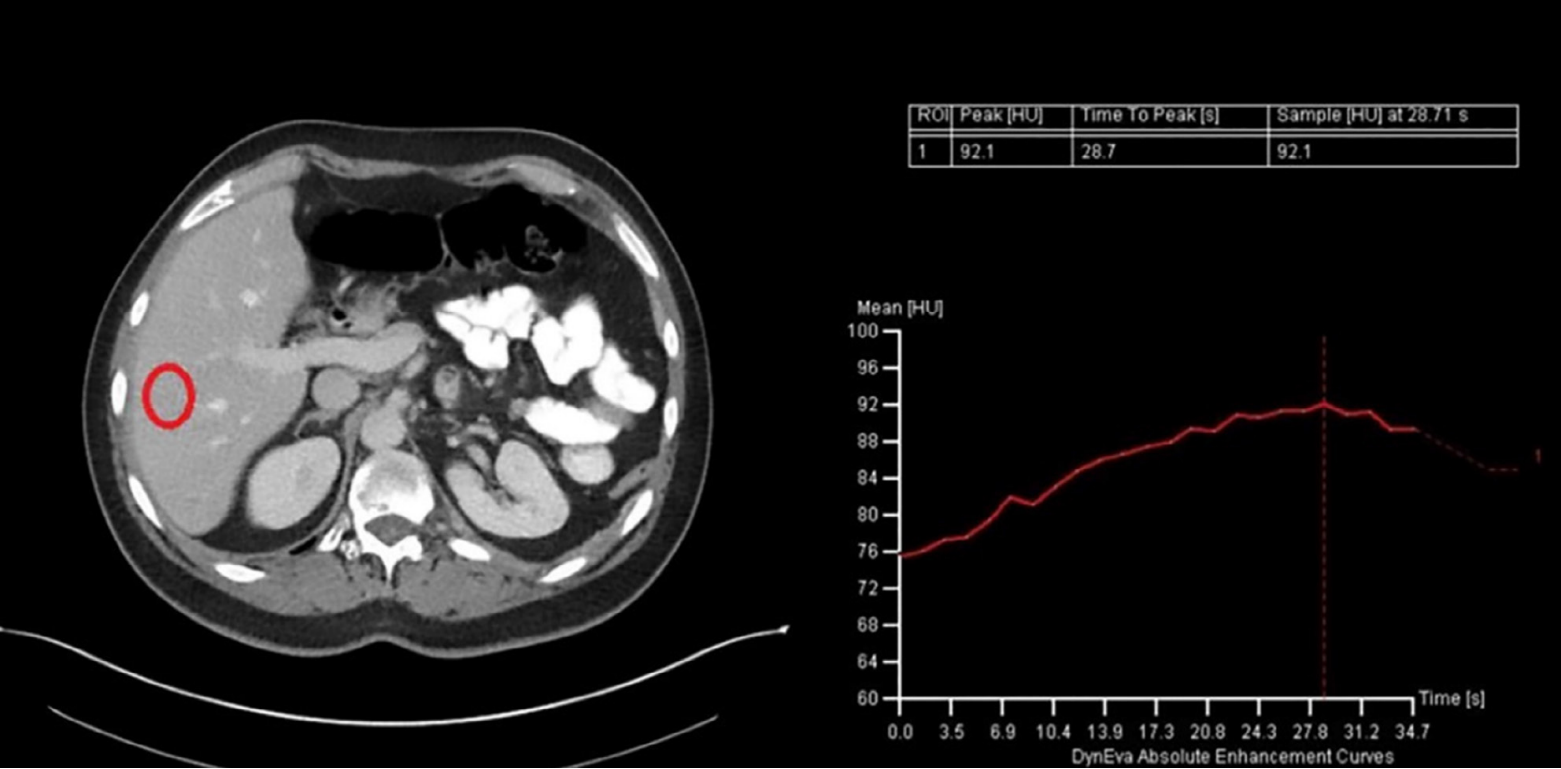
Example of a portal venous phase scan at the location of the hepatic hilum of the vena porta and the corresponding HU‑curve from HU measurements in an ROI that was selected for manual triggering.

Manual triggering in 100 cases showed an average time to peak or plateau enhancement of 102 s, which is significantly different from 90 s. Figure 3 shows an example of a portal venous phase scan with manual triggering using HU measurements in an ROI at the hilus of the vena porta. Patient weight significantly influenced the timing of peak enhancement. A formula to predict the interval time between injection and peak enhancement or plateau was:


$$\begin{array}{c} Time\;to\;plateau/peak\;enhancement\;(s)\\ \;=66\;+\;0.48*weight\;(kg). \end{array}$$ Eq. 5

## Discussion

This study focuses on three critical aspects of optimizing iodinated contrast media injection protocols for abdominal CT: establishing a target HU value, developing a formula for patient‑specific contrast agent volumes and determining a timing for achieving peak tissue enhancement. This study has addressed limitations in current practices by providing a robust, evidence‑based foundation for personalized imaging protocols.

The target HU value was derived from retrospective data and calculated as the 25th percentile of observed HU values plus a safety margin. This study had used the mean values of portal venous phase HU enhancement in seven tissues, but could be generalized towards specific tissues, such as the liver.

Current approaches to achieving target HU values often rely on fixed contrast volume dosages or empirical adjustments. Our study, on the contrary, emphasizes the importance of tailoring enhancement thresholds to specific clinical needs and patient characteristics, and the formula is no longer a proprietary equation of the injection device.

In the current study, it was aimed for a constant target HU value of 125. However, regression could also be performed towards higher HU enhancement levels for thinner patients or lower HU values for obese patients. Regression analysis would have to be calculated for adjusted ideal volumes. Our methodology facilitates these adjustments, ensuring optimal imaging for diverse scenarios [21, 22].

Manual determination of HU values was a cornerstone in the present study. However, leveraging artificial intelligence (AI) techniques, such as convolutional neural networks, presents an opportunity to automate this process [23]. Automation could streamline optimization or verify the quality of the HU enhancement values. For instance, automated tools could evaluate enhancement in real time, alerting clinicians to suboptimal imaging and enabling immediate corrective measures. This innovation bridges the gap between research and clinical practice, making advanced imaging protocols more accessible.

Our study revealed an average time to plateau or peak enhancement of 102 s, which is slightly longer than the conventional 90 s delay commonly used in our hospital since the 1980s. This modest extension accounts for advances in CT technology and variable contrast agent volumes, highlighting the need for adaptive timing strategies. Interestingly, while the patient’s weight influenced enhancement timing, other parameters (age, gender, and height) showed only a negligible effect. The impact of the weight is, however, limited and probably linked with the larger contrast agent volumes that are all injected at the same injection rate of 1.5 ml/s. Patients with larger weight or height may therefore have a peak enhancement later in time.

Importantly, the role of timing in enhancing imaging outcomes becomes more critical in applications such as tumor density measurement for colorectal cancer and neuroendocrine liver metastases. By initiating scans precisely at the peak enhancement moment, a better visualization of vascular structures, tissues, and lesions will occur, which may translate into enhanced diagnostic accuracy. However, this was beyond the scope of the current study.

Our findings on the impact of weight, height, and heart rate on the ideal contrast agent volumes align with existing research [11], underscoring the relevance of these parameters and/or the lean body weight for personalized injections. Moreover, the flexibility of our equation allows clinicians to adjust contrast agent volumes for different imaging objectives, such as reducing iodine exposure in low‑kilovolt protocols or adapting volumes for spectral or mono‑energetic CT.

The findings of this study extend beyond conventional CT to applications like dual‑energy CT and mono‑energetic reconstructions. By applying energy‑specific correction factors, the proposed methodology can optimize imaging across diverse settings, ensuring consistent quality irrespective of scanner type or protocol. This is related to the predictable or measurable HU values of the same amounts of iodine under different scan conditions.

### Limitations and future directions

While this study offers a robust framework for contrast agent optimization, several limitations warrant consideration. First, the findings are based on data from a single institution, albeit using four different CT scanners. Expanding the research to diverse populations and multiple centers would enhance its generalizability. Second, while the proposed formula was validated using retrospective data, prospective studies are necessary to confirm its clinical efficacy. Similarly, the timing recommendations require further validation in routine practice, as manually triggered venous phase scans are not standard.

Additional research could explore more sophisticated statistical methods or incorporate other physiological factors that may influence contrast distribution.

## Conclusion

We have proposed a regression‑based method to develop new equations for personalized injection volumes and have applied this method for venous phase enhanced abdominal CT. In combination with a target HU and newly measured time to peak enhancement, a comprehensive injection protocol can now be introduced.
